# Actually Seeing What Is Going on – Intravital Microscopy in Tissue Engineering

**DOI:** 10.3389/fbioe.2021.627462

**Published:** 2021-02-17

**Authors:** Ravikumar Vaghela, Andreas Arkudas, Raymund E. Horch, Maximilian Hessenauer

**Affiliations:** Department of Plastic and Hand Surgery, University Hospital of Erlangen, Friedrich–Alexander University Erlangen–Nürnberg (FAU), Erlangen, Germany

**Keywords:** tissue engineering, intravital microscopy, leukocyte recruitment, biomaterial, fluorescence, *in vivo*

## Abstract

Intravital microscopy (IVM) study approach offers several advantages over *in vitro*, *ex vivo*, and 3D models. IVM provides real-time imaging of cellular events, which provides us a comprehensive picture of dynamic processes. Rapid improvement in microscopy techniques has permitted deep tissue imaging at a higher resolution. Advances in fluorescence tagging methods enable tracking of specific cell types. Moreover, IVM can serve as an important tool to study different stages of tissue regeneration processes. Furthermore, the compatibility of different tissue engineered constructs can be analyzed. IVM is also a promising approach to investigate host reactions on implanted biomaterials. IVM can provide instant feedback for improvising tissue engineering strategies. In this review, we aim to provide an overview of the requirements and applications of different IVM approaches. First, we will discuss the history of IVM development, and then we will provide an overview of available optical modalities including the pros and cons. Later, we will summarize different fluorescence labeling methods. In the final section, we will discuss well-established chronic and acute IVM models for different organs.

## Introduction

*In vitro* study models have immensely endorsed our knowledge of cellular physiology. *In vitro* study models hold many advantages over *in vivo* study models. Cells can be isolated from the particular organ, manipulated and propagated as per the requirement of the study (Kapałczyńska et al., 2018; Chen et al., 2019). Simplicity and low-cost maintenance requirements make 2D culture the first choice for researchers to understand cell biology, tissue development, disease mechanisms, and drug development. 2D culture studies are been used for better understanding of cancer biology, vascular development, cell secretomes and their influences on the immediate environment (Kapałczyńska et al., 2018; Al-Abboodi et al., 2019; Kengelbach-Weigand et al., 2019; Ahmadzadeh et al., 2020). However, *in vitro* 2D models fail to mimic the native tissue environment which is important to study tissue physiology (Weigand et al., 2016; Duval et al., 2017; Kapałczyńska et al., 2018). Therefore, 3D models were designed to propagate the cells in a more native tissue environment (Witt et al., 2017; Tong et al., 2018; Kengelbach-Weigand et al., 2019). Many researchers are shifting from traditional 3D models to biofabricated 3D models which facilitates replication of the complex tissue architecture in a more precise and controlled manner (Horch et al., 2018). Though, most 3D models lack other influencing factors of native tissue, such as the presence of other cell types as well as signal molecules from the immediate and distant environment (Weigand et al., 2016; Kapałczyńska et al., 2018). Hence, conducting *in vivo* studies is required to overcome above-mentioned limitations. A typical *in vivo* study ends with the killing of the animal followed by a collection of an organ of interest. The histological analysis provides information at one static point which fails to describe the dynamics of ongoing cellular processes (Steiner et al., 2019).

At this point, IVM studies excel *in vitro, ex vivo* and 3D models. IVM provides imaging of cellular events in its native tissue environment as well as in real-time setting. It can be used to examine proliferation, migration, differentiation of cells as well as their specific interactions and behavior (such as leukocyte–endothelial, tumor cell–cell, and bacterial-cell interaction). IVM enables acute and/or chronic as well as repetitive imaging in the same animal, which provides a comprehensive picture of the overall complex dynamic processes. IVM provides an opportunity to analyze different stages of tissue regeneration simultaneously.

Various optical modalities, ranging from wide field to multiphoton microscopy, are available for imaging of the targeted organ (Wang et al., 2005; Wang H. et al., 2018; Jonkman and Cm, 2015). The conventional wide-field microscope is adequate for semitransparent tissue structures such as cremaster muscle and skin (Lemaster et al., 2017). Confocal microscopy can further increase the resolution. Additional deep imaging of complex organs can be achieved with improved optic modalities such as multiphoton microscopy (Theer et al., 2003; Horton et al., 2013; Weigert et al., 2013; Ouzounov et al., 2017). Combination of appropriate microscopic modality and genetic tools or contrast agents can be applied to understand specific organ physiology via IVM.

Earlier IVM studies were restricted to acute duration. However, the advent of window and chamber models helped to elongate the experimental period. IVM window models provide further benefits such as elimination of repeated surgical preparation and observation of the same region for multiple times in the same animal (Hackl et al., 2013; Reichel et al., 2015; Hessenauer et al., 2018). Apart from that, biomaterials play an important role in modern tissue engineering. Tissue engineered scaffolds serve to replace, repair, and maintain structural integrity of tissue. Scaffolds should be biocompatible and promote cell growth and differentiation to support regeneration (Patel and Fisher, 2008). IVM is a promising and fast approach to study interactions of different tissue engineered constructs for tissue development. Overall, it enables tracking of the entire dynamic process. Above mentioned advantages reduce the inter-animal variation and overall requirement of the number of animals (Prunier et al., 2017).

Considering all the advantages of IVM, it is indispensable to discuss various aspects of IVM. It is a need of an hour to combine advanced optical modalities and fluorescence tagging methodologies and apply them in IVM for an in-depth analysis of the healthy and diseased state of the tissue, tissue development, repair and biocompatibility as well as host reactions on implanted biomaterials. Therefore, in this review, we aim to begin with a short history of IVM development, followed by an overview of available optical modalities and contrast agents. In the final section of the review, we will discuss well-established IVM models for different organs.

## History

In the early 19th century, Rudolf Wagner for the first time reported rolling leukocytes in the blood vessel of a grass frog. This was one of the earliest report involving real-time observation of vascular physiology in the alive animal. But the roots of IVM are even deeper. The Italian scientist Marcello Malpighi attempted IVM to observe the lung in mammals as well as amphibians for the very first time in the 16th century. In the late 19th century, Elie Metchnikoff studied phagocytosis and diapedesis using IVM in frog. The earliest IVM movies were created in the early 20th century by Ries and Vles. Before that, drawing was the only tool to describe the observation. Until then, IVM imaging was limited to vasculature observation employing bright-field microscopic setup. Moreover, observation and documentation were difficult due to the lack of contrast agents (Secklehner et al., 2017).

Intravital microscopy became a more practical tool for physiological studies after the introduction of the first fluorescence microscope by Heimstadt in 1911 (Secklehner et al., 2017) and after the development of exogenous fluorophores. In 1955, the confocal scanning microscope was developed by Minsky (1988). It was designed to eliminate out-of-focus emission light with the help of pinhole. Confocal microscopy also enhances contrast and improves Z-resolution (Wang et al., 2005; Jonkman and Cm, 2015).

Physicist Maria Göppert-Mayer in 1931 introduced the idea of multiphoton microscopy. However, the application of multiphoton microscopy became only possible after the development of the required excitation lasers in 1976. Multiphoton microscopy works on the principle of simultaneous absorption of two or more photons. Advantages of multiphoton microscopy include deeper tissue penetration and lower phototoxicity. Advanced optical modalities along with newly developed window or chamber models open the door for longitudinal deep-tissue imaging (Schießl and Castrop, 2016).

The advent of fluorescent protein and fluorescent probes has played an important role in imaging. In 1994, Green fluorescent protein (GFP) originally isolated from Aequorea victoria, was successfully introduced into *Caenorhabditis elegans* as a genetic marker. 3 years later, first strains of GFP transgenic mice was reported. Later on, different fluorescent proteins such as red, yellow, and cyan fluorescent proteins (RFP, YFP, and CFP) were discovered (Hadjantonakis et al., 2003). On the other hand, application of the first fluorescently labeled antibody was already reported in 1942 by Albert Coons (Zanacchi et al., 2014).

Transgenic reporter animals, fluorescent probes, window models and advanced microscopic modalities have emerged as essential IVM tools to study target tissues at a cellular level. The development of window models is particularly useful for chronic experiments. In 1924, Sandison first used a transparent chamber in the rabbit’s ear. Currently, organs such as skin, liver, kidney, lung, cremaster muscle and brain have been studied using window models. Since the mid-20th century, researchers are actively using this tool for physiological research (Secklehner et al., 2017).

## Microscopy Techniques

The journey of IVM started with bright field transillumination microscopy where the image is formed by the light transmitted through the sample (Pittet and Weissleder, 2011). However, transillumination is not suitable for relatively dense and thick tissues. Therefore nowadays, IVM is largely based on the epi-fluorescence principle where the image is generated from the fluorescence emitted from the object (Weigert et al., 2013). Several microscopic modalities are available for performing IVM such as wide-field fluorescence, confocal and multiphoton microscopy (Table 1). When light is absorbed by the fluorophore, electrons are excited from the ground state to the excited state. While returning to the ground state, electrons emit light which has a longer wavelength. This emitted light is collected in a detection system and generates fluorescence image. In wide-field microscopy, the entire field of view is illuminated. Here, the detection of out of focus light compromises the resolution of an image (Swedlow et al., 2002; Weigert et al., 2013).

**TABLE 1:** Imaging techniques (Mempel et al., 2003; Masedunskas et al., 2012; Weigert et al., 2013; Marques et al., 2015b; Vielreicher et al., 2017).

Excitation	Technique	Microscopy	Light source	Detection	Advantages	Disadvantages
Single-photon	Widefield		Mercury lamp/LED	CCD	• Fast acquisition • Low cost	• Limited depth • Phototoxicity
		RLOT	Xenon lamp	CCD	• Detection of fast dynamic activity in the absence of specific fluorophores	• Limited to translucent tissue
	Confocal	LCSM	Lasers	PMT	• High spatial resolution • 3D sectioning	• Limited depth • Slow acquisition • Phototoxicity • Relatively high cost and smaller field of view
		SDCM	Lasers	CCD	• Fast acquisition • Low phototoxicity • 3D sectioning	• Limited depth • Faster acquisition • Pinhole crosstalk reduces the resolution
Two or more photon	Multiphoton	Two-photon	Lasers	PMT	• Extended depth • No off-focus emissions	• High cost • Slow acquisition
		Three-photon	Lasers	PMT	• Deep tissue imaging • Improved signal to background ratio	• High cost • Slow acquisition
		SHG and THG	Lasers	PMT	• No energy absorption • Label-free imaging of collagen, myosins, myelin, and lipids	

*LED, light-emitting diode; RLOT, reflected light oblique transillumination; CCD, charge-coupled device; LCSM, laser confocal scanning microscopy; PMT, photomultiplier tube; SDCM, spinning disk confocal microscopy; SHG, second-harmonic generation; THG, third-harmonic generation.*

This problem is resolved in confocal microscopy (White et al., 1987; Minsky, 1988). In laser confocal scanning microscopy (LCSM), the focus light is removed by the introduction of a pinhole in front of the photomultiplier detector. The specimen is scanned point-by-point. Scanned images of each depth can be combined to form a 3D image. However, scanning of all the focal plane makes the image acquisition slower and poses a phototoxicity issue. Image acquisition speed can be increased using multiple pinholes in spinning disk confocal microscopy (SDCM). Therefore, SDCM reduces phototoxicity (Wang et al., 2005; Jonkman and Cm, 2015; Bai et al., 2020).

In multiphoton microscopy (MPM) two or more photons having near-infrared wavelength are absorbed simultaneously. Fluorophore excitation takes place only at the in-focus plane, which reduces its phototoxicity and eliminates the requirement of pinholes. MPM is preferred when the imaging area is located more than 50–100 μm deep in the tissue (Weigert et al., 2013). Two photon microscopy (2 PM) can reach up to superficial cortical layers of the rodent brain (Miller et al., 2017). Light scattering and absorption of the tissue limit the penetration depth of 2 PM. Scattering and absorption both are dependent on excitation wavelength (Miller et al., 2017). However, recent developments in Three PM (3 PM) have demonstrated substantial improvement in penetration depth. Horton et al. (2013) first used 3 PM at the long-wavelength window of 1,700 nm for mouse brain imaging. 3 PM has emerged as a powerful game-changer in high-resolution, deep tissue intravital imaging. 3 PM enables imaging of vascular and neuronal structures at the depth of approximately 1.3 mm in the mouse brain (Horton et al., 2013).

Other available MPM variations include second and third harmonic generations (SHG and THG). SHG and THG provide label-free visualization of structures, such as collagen, myosin, and lipids (Reichel et al., 2015; Vielreicher et al., 2017). The signal is generated when two or more photons combine and form single photon without energy loss. SHG and THG enable 200–400 μm of imaging depth (Weigert et al., 2013).

Reflected light oblique transillumination (RLOT) microscopy works on the principle of oblique transillumination. It was developed by installing reflector directly below the specimen. The tilted reflector allows only a specific diffracted sideband of light to reach the objective lens. It can be incorporated with a wide-field epi-fluorescence microscope. RLOT can be used for imaging fast dynamic activity in the absence of specific fluorophores (Mempel et al., 2003).

## Stainings/Probes Used in Intravital Microscopy

Most tissues are complex structures made up of different function-specific cells. Therefore, it is very important to study all cell types discretely. Using IVM alone, it can be difficult to differentiate between different tissue-specific cell types. It is important to distinguish the target via tagging or injection of contrasting dye in the animal. This can be achieved by application of fluorescence dyes, cell-specific labeling using antibodies, nanotechnology-based probes and use of genetic reporters. Some of the dyes are already being used for clinical purpose (Dunn and Ryan, 2017; Ludolph et al., 2019).

The discovery of fluorophores in conjugation with biologically active substances (peptides, antibody fragments, and nanoparticles) led to major advancements in IVM. Depending on the requirement of the study, fluorophores such as TRITC or FITCs can be conjugated to high or low molecular weight molecules such as Dextran or Albumin. TRITC or FITCs in conjugation with high molecular weight Dextran is commonly used for contrast enhancement of intravascular blood plasma. FITC conjugated to lower molecular weight Albumin easily leaks out from the endothelium, therefore it is used in plasma extravasation studies. Injectable fluorophores have played important role in studying biological processes such as leukocyte trafficking, cell–cell interaction, including inflammation, angiogenesis, apoptosis, oxidative stress, and calcium dynamics (Dunn et al., 2002; Taqueti and Jaffer, 2013; Kawakami, 2016).

Genetically encoded fluorescent proteins (FPs) are one of the most preferred approach amongst researches for *in vivo* imaging. Genetic integration and exemption of substrates or cofactors for fluorescence make FPs an ideal tool for IVM. Available FPs enable cell tracking and *in vivo* proliferation during development, tumors metastasis and in stem cells therapy models. Far-Red fluorescent proteins (RFPs) are preferred over GFPs due to lower light absorption by hemoglobin which allows efficient photon transmission and less autofluorescence (Taqueti and Jaffer, 2013). Taqueti and Jaffer (2013) used ApoE^–/–^/Lysozyme ^EGFP/EGFP^ mice containing encoded GFP neutrophils and monocytes to study leukocyte trafficking. Looney et al. (2011) used c-fms EGFP transgenic mice for lung immune surveillance. Lee et al. (2014) used Cxcr6gfp/+mice to study NK T cells in the liver vasculature during Borrelia burgdorferi infection. Fuhrmann et al. (2010) studied Alzheimer’s disease-linked neuron loss in microglial Cx3cr1 knockout mice (Kawakami, 2016). Similarly, RFP and YFP have been used to study various immune as well as organ-specific cells (Table 2). Genetic cell labeling enables discrimination between metastatic and non-metastatic tumors cells (Condeelis and Weissleder, 2010; Taqueti and Jaffer, 2013; Kawakami, 2016). However, the considerable size of FPs (∼25–30 kDa), can interfere with protein function. Moreover. FPs exhibit low brightness and photostability (Toseland, 2013; Yan and Mp, 2015).

**TABLE 2:** Fluorescence probes for intravital microscopy (Condeelis and Weissleder, 2010; Jin et al., 2011; Taqueti and Jaffer, 2013; Toseland, 2013; Caravagna et al., 2016; Kawakami, 2016; Wang H. et al., 2018).

Class	Subtypes	Examples/target
**Fluorescence dyes**		
	TRITC-dextran, FITC-dextran Texas red-dextran	Vascular contrast enhancement, plasma extravasation
	FITC-albumin	Plasma extravasation
	Rhodamine 6G, Acridine orange	Leukocyte trafficking
	Hoechst 33342	DNA staining
	CMTMR, Calcein-AM, CFSE, CMAC	*Ex vivo* cell labeling
**Genetic tags**	**GFP**	
	Lysozyme-EGFP	Neutrophils and monocytes
	c-CSF1R-GFP	Neutrophils, monocytes macrophages
	CX3CR1-GFP	Monocytes, macrophages, microglia
	CXCR6-GFP	NK T cells
	**RFP**	
	CX3CL1-Cherry	Macrophages
	CD2-RFP	T cells
	IL17f-RFP	Th17 cells
	NG2-RFP	Pericytes
	tdTomato	HA-CTLs
	**YFP**	
	CD11c-EYFP	Dendritic cells
	Thy1-YFP	Neuron
**Antibodies**	**IgG**	
	EGFR, Her2/neu, c-MET	Tumor cells
	CD31/PECAM-1	Endothelial junctions
	ICAM-1	Endothelial cells, leukocytes
	VCAM-1	Endothelium
	CD45	Pan-leukocyte
	CD11b	Myeloid leukocytes
	Ly-6G	Neutrophils
	F4/80	Monocytes, macrophages
	GPIbβ	Platelets
	**Fragments**	Fab, diabody, minibody
**Nanotechnology**	***Q-dot***	
	CNA35-QD525	Inflammation
	CdTe/CdS	Vascular imaging
	**Magnetic nanoparticles**	
	CLIO-AF555, CLIO-VT750	Macrophages
	cRGD-CLIO(Cy5.5), scrRGD-CLIO(Cy3.5)	Tumor cells

*TRITC, tetramethylrhodamine; FITC, fluorescein isothiocyanate; CMTMR, 5-(and-6) -(4-chloromethyl (benzoyl)amino) tetramethylrhodamine; CFSE, carboxyfluorescein succinimidyl ester; CMAC, 7-amino-4-chloromethylcoumarin; EGFP, enhanced green fluorescent protein; GFP, green fluorescent protein; CSF1, colony-stimulating factor 1; CX3CR1, C-X3-C motif chemokine receptor 1; CX3CL1, C-X3-C motif chemokine ligand 1; CD, cluster of differentiation; RFP, red fluorescent protein; IL17f, interleukin 17f; NG2, neuron glia antigen-2; EYFP, enhanced yellow fluorescent protein; Her2/neu, human epidermal growth factor receptor 2; PECAM-1, platelet endothelial cell adhesion molecule; ICAM-1, intercellular adhesion molecule 1; Ly6G, lymphocyte antigen 6 complex locus G6D; CNA35, collagen-binding adhesion protein 35; CdS/CdTe, cadmium sulfide/cadmium telluride; CLIO, crosslinked iron oxide; AF555, AlexaFluor555; VT750, VivoTag-S 750; cRGD, cyclic arginine-glycine-aspartic acid peptide; scrRGD, scrambled RGD; Cy, cyanine.*

Another way to detect specific cell types in IVM is by using fluorescently labeled antibody against specific cell receptor. Several types of antibody-based markers are developed to specific tagging of cells. Fluorescently labeled antibody human epidermal growth factor receptor type 2 (HER2)/*neu*, epidermal growth factor receptor (EGFR) and c-MET have been used to study tumor growth (Tanaka et al., 2014). Endothelial cells can be targeted using an anti CD31 antibody. During migration endothelial cells and leukocytes express Intracellular adhesion molecule (ICAM)-1 and endothelial cells express vascular cell adhesion molecule (VCAM)-1. Antibodies against such adhesion molecule can be used to study vascular cell migration. Apart from using full antibodies, Fluorophore-conjugated antibody fragments (Fab, Diabody, and Mini body) can also be used for IVM (Condeelis and Weissleder, 2010; Taqueti and Jaffer, 2013).

Conventional fluorescent organic dyes and FPs have limitations of photobleaching, low signal intensity, and spectral overlapping (Wang H. et al., 2018). These limitations can be overcome via the application of nanotechnology-based probes known as Quantum dots (QDs). QDs show unique properties such as size-tunable light emission, high signal brightness, extended photostability and resistance against metabolic degradation, simultaneous multi-color excitation, and spectral multiplexing (Resch-Genger et al., 2008; Jin et al., 2011; Shao et al., 2011). Megens et al. (2010) used collagen-binding protein labeled with green-fluorescent quantum dots (CNA35- QD525) to study subendothelial collagen. Wang H. et al. (2018) developed mercapto succinic acid (MSA) capped cadmium telluride/cadmium sulfide (CdTe/CdS) QDs for long-term vascular IVM. Ripplinger et al. (2012) used magnetofluorescent nanoparticles (MFNP) such as cross-linked iron oxide (CLIO) AF555, CLIO-VT680 to illuminate macrophages during inflammation. Montet et al. (2006) used cRGD-CLIO(Cy5.5) and scrRGD-CLIO(Cy3.5) for imaging tumor cells. Similarly, Mulder et al. (2009) used RGD-pQDs for targeted imaging of tumor angiogenesis. Biocompatibility and specificity of QDs can be modulated by surface coating modification. However, potential toxicity poses uncertainty for the *in vivo* application of QDs. Cytotoxicity of QDs depends on factors such as charge, size, coating ligands, oxidative, photolytic, and mechanical stability (Resch-Genger et al., 2008; Jin et al., 2011; Shao et al., 2011; Progatzky et al., 2013).

## Models/Operation Techniques

Over the past decades, different window and chamber models have been developed according to the location of the organ of interest. Most models required surgical procedures to expose the organ of interest and installation of window or chamber. In this section, we will discuss various IVM models. Depending on study duration, IVM models can be divided into acute imaging models and chronic models.

### Acute IVM Models

In acute models, the desired organ or tissue is surgically exposed for a short period and the animal is sacrificed at the end of the study. IVM is limited to a specific time point and repeated observation is unattainable.

#### Cremaster Muscle

**FIGURE 1:**
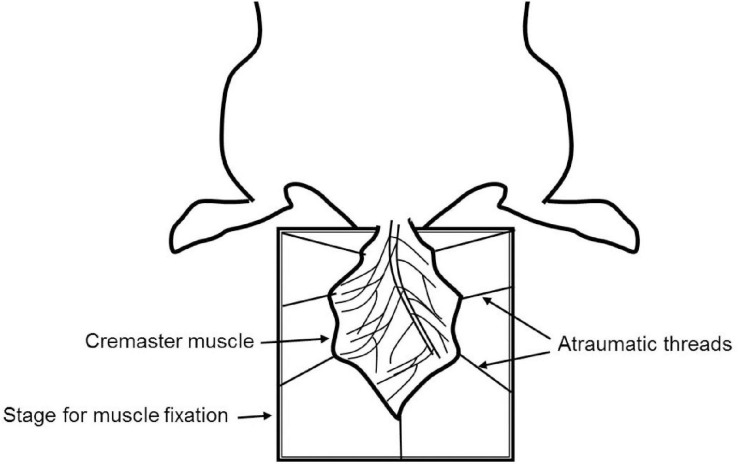
Schematic representation of cremaster muscle model for IVM.

The cremaster muscle is a very thin and nearly transparent layer of smooth muscle covering both testicles. It is easily accessible in male rodents via a minimally invasive surgical procedure, which allows high-resolution imaging of local the microvasculature (Figure 1).

The cremaster muscle is surgically exposed by a longitudinal incision of the scrotum. After freeing from the surrounding connective tissue, the apex of the cremaster muscle is fixed on a customized stage for superfusion. A longitudinal incision is made through the ventral surface of the muscle followed by detachment from epididymis and testicle. The testicle is either pushed back into the abdominal cavity or removed by orchiectomy. The remaining cremaster muscle is spread over the customized stage and can be accessed for microscopy and interventions (Bagher and Segal, 2011; Reichel et al., 2011; Donndorf et al., 2013).

This well-standardized surgical procedure can be a useful tool for visualizing and analyzing capillary perfusion, leukocyte–endothelial interaction, microvascular response to different stimuli and endothelial permeability in a defined environment (Donndorf et al., 2013; Molski et al., 2015). It can also be used to study blood cell interactions under influence of different drugs and chemokines (Reichel et al., 2012; Rius and Sanz, 2015) as well as ischemia-reperfusion (IR) injury and local effects of systemic conditions (Molski et al., 2015). Cremaster muscle is an acute IVM model. However, Siemionow and Nanhekhan (1999) developed a chronic cremaster chamber which allows imaging up to 3 days.

#### Heart

The heart is the essential blood pumping machinery of the body. Therefore, it is very important to understand heart physiology. Application of IVM provides more accurate information compared to *in vitro* or *ex vivo* setting as it does not mimic the native physiological environment (Vinegoni et al., 2015). Similar to the lung, continuous movement is a major obstacle in heart IVM. Physical immobilization such as a using suture, a mechanical stabilizer or suction can be applied but they end up in low-resolution imaging and movement artifacts (Aguirre et al., 2014). To overcome this, a combination of approaches such as a gated acquisition algorithm, gated sequential segmented microscopy or active motion stabilization along with mechanical stabilization has been used (Lee et al., 2008).

For IVM, thoracotomy in the fourth left intercostal space is performed. Surrounding connective tissue is removed to expose the heart. Once exposed, the heart is stabilized using one of the above-mentioned technique (Lee et al., 2012; Aguirre et al., 2014; Vinegoni et al., 2015).

Heart IVM can be used to study both function of the heart muscle on single-cell level within the muscle with regard to cell metabolism as well as cell electrophysiology under physiological and pathophysiological conditions. In addition cell dynamics in pathological conditions such as ischemia-reperfusion and myocardial infarction can be closely monitored (Aguirre et al., 2014; Vinegoni et al., 2015). Most importantly microvascular events such as leukocyte trafficking as well as microvascular rearrangement under many pathological conditions such as myocardial infarction or infection can be evaluated (Ueno et al., 2016; Matsuura et al., 2018; Bajpai et al., 2019).

#### Ear Pinna Model

**FIGURE 2:**
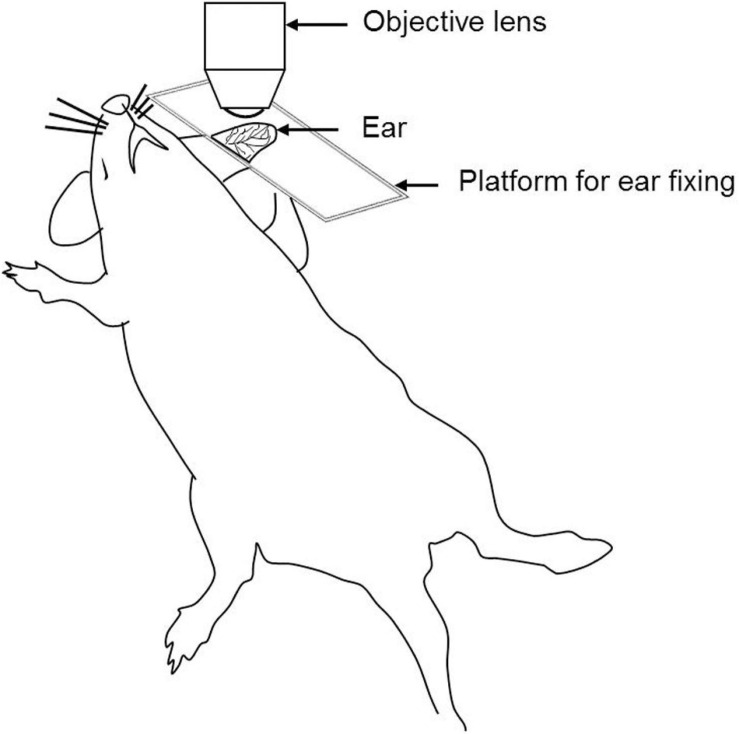
Schematic illustration of the ear pinna model for IVM.

The ear pinna model is the easiest model for *in vivo* imaging as it is a non-surgical procedure (Figure 2). The ear contains two full-thickness layers of skin separated by a thin cartilage layer. The skin of the mouse ear contains few hair and closely resembles human skin (Chan et al., 2013). Ease of access, minimal preparation requirements, and less respiratory movement makes it an ideal site for the investigation of cell migration dynamics, cell–cell and host cell–pathogen interactions (Secklehner et al., 2017). It is very important to remove the small number of hairs present on the ear. Otherwise, it may cause autofluorescence during imaging. Shaving of the ear hair can easily lead to skin damage. Moreover, it provides only a limited area for IVM (Chan et al., 2013; Strüder et al., 2017).

The rodent ear is a suitable to model for investigating immune cells in the skin, tissue implantation, and *in vivo* tumor cell behavior (Chan et al., 2013), IR injury, and wound healing (Chan et al., 2013; Strüder et al., 2017).

#### Salivary Gland

Membrane traffic is a fundamental transport process that encompasses the exchange and distribution of molecules such as proteins, lipids, and polysaccharides between the cell and the extracellular space as well as among intracellular organelles (Ebrahim and Weigert, 2019). The salivary gland has emerged as a revolutionary acute IVM model to study endocytosis and regulated exocytosis. The salivary gland is situated in the neck region which makes it less susceptible to motion artifacts created by respiration and heart beating (Masedunskas et al., 2013b; Ebrahim and Weigert, 2019). The salivary gland can be easily accessed by removing a small circular piece of skin from the neck (Masedunskas et al., 2013a). Relatively easy surgical access and ease of selective manipulation make it an excellent IVM model (Ebrahim and Weigert, 2019).

This model has been successfully applied to investigate mitochondrial dynamics (Porat-Shliom et al., 2019), endocytosis mediated remodeling as well as endocytosis modulation in cancer progression (Milberg et al., 2017; Ebrahim and Weigert, 2019).

### Chronic IVM Models

Chronic IVM models are primarily designed for both longitudinal as well as acute studies. Chronic models involve surgical preparation along with installation of a window or chamber that enables rapid and long-term imaging.

#### Kidney

The kidney is a complex organ. It contains more than 20 function-specific cell types. The kidney contains several nephrons which are responsible for glomerular filtration, active tubular secretion as well as reabsorption of useful molecules (van den Berg et al., 2018). The kidney is exposed by a flank incision through the retroperitoneum. IVM is performed by placing the kidney in a coverslip-bottomed cell culture dish or immobilizing it by custom made holder (Dunn et al., 2007; Hato et al., 2018). Although, kidney IVM is primarily an acute model, Hackl et al. (2013) demonstrated the modified method involving of repeated externalization of the kidney which enables *in vivo* multiphoton imaging over several days.

Kidney IVM can be used to investigate a renal injury, IR injury, dysfunction, inflammation, cell death, microvascular blood flow, glomerular filtration and podocyte migration (Russo et al., 2007; Devi et al., 2013; Hackl et al., 2013; Hall et al., 2013; Schießl et al., 2016b).

#### Lung

The lung is an essential respiratory organ situated below the rib cage. It contains a unique capillary network. Leukocytes need to undergo shape deformation for traveling through the narrow capillary segments (Wiggs et al., 1994). Pioneering work on acute imaging of lung was done by the Presson group where the dog was use as model organism. Later, this model was developed for small animals (Presson et al., 1994). Continuous movement caused by breathing and heart beating poses difficulties for *in vivo* imaging. Presson et al. (2011) for the very first time applied a customized vacuum ring imaging window with adjunctive support of gated imaging or frame registration for efficient reduction of motion artifact and maximizing clarity of the image. This organ stabilization approach revolutionized IVM in organs which are susceptible to motion artifact. Several other approaches have been used to stabilize lung for IVM which includes mechanical stabilization using Bronchus clamping, glue fixation on a coverslip and suction stabilization. However, immobilization of one area of the lung can induce shear force which can injure the lung (Looney et al., 2011; Fiole and Tournier, 2016; Rodriguez-Tirado et al., 2016).

For exposing the lung, thoracotomy is performed. The animal is placed in the right or left lateral decubitus position. An incision is made to expose the rib cage. A couple of ribs (3–4) are removed to expose the surface of a lung lobe followed by stabilization for microscopy (Looney and Bhattacharya, 2014; Rodriguez-Tirado et al., 2016). Using efficient optical tools, high-resolution lung imaging can be performed for up to 12 hours (hrs) (Rodriguez-Tirado et al., 2016). However, recently, Entenberg et al. (2018) made a ground breaking success by developing a permanently implantable and minimally invasive window that can be imagined for up to 2 weeks.

Lung IVM can be applied to study mitochondrial function in lung immunity, neutrophil as well as platelet trafficking, the gas exchange process and lung tumor biology (Eichhorn et al., 2002; Tabuchi et al., 2008; Kreisel et al., 2010; Fiole and Tournier, 2016; Rodriguez-Tirado et al., 2016; Thanabalasuriar et al., 2016; Yipp et al., 2017; Neupane et al., 2020). Apart from lung wobbling and surgical invasiveness, a major problem in the lung is penetration depth. Even highly efficient two-photon microscopy can only image superficially (up to 30–100 μm), which may not display deep tissue features of the lung (Perlman and Bhattacharya, 2007; Looney et al., 2011).

#### Spleen

The spleen is an important secondary lymphoid organ for IVM. It is located below the rib cage on the left-hand side within the abdominal cavity. The spleen filters pathogens and antigens from the blood. It contains the red pulp and white pulp regions, separated by the marginal zone. Red pulp macrophages recycle iron from senescent erythrocytes. The white pulp contains T cell and B cell zones which are important for antigen-specific immune responses (Martin-Jaular et al., 2011). It is relatively easy to prepare spleen for IVM because of its superficial location in the body (Secklehner et al., 2017).

To expose the spleen, an incision is made below the ribcage on the left lateral position. Afterward, the spleen is exteriorized and placed on a customized stage and sealed with adhesive (Ferrer et al., 2012; Deniset et al., 2017). Spleen IVM is utilized in disease models such as malaria (Secklehner et al., 2017), atherosclerosis (Robbins Clinton et al., 2012) as well as cancer (Cortez-Retamozo et al., 2012). Furthermore, it can also be used for imaging of lymphocytic Calcium ion signaling (Yoshikawa et al., 2016).

#### Liver

The largest metabolic organ liver is located below the diaphragm. It plays an essential role in metabolism, protein synthesis and detoxification of systemic circulation (Vollmar and Menger, 2009). It receives around 80% blood supply from the portal vein and the remaining 20% oxygenated blood from the hepatic artery. Hepatocytes are the most abundant cell types inhabiting liver. Apart from that, it also contains sinusoidal Kupffer cells, endothelial cells, stellate cells and lymphocytes (Vollmar and Menger, 2009; Marques et al., 2015a).

As per the requirement of the study, liver IVM can be performed starting from a few hours to days (Ritsma et al., 2012; Park et al., 2018). The surgical procedure involves the opening of the abdominal cavity. A small part of right liver lobe is carefully exteriorized and placed on a handcraft stage (Marques et al., 2015b). Long term IVM requires installation of an observation window in the abdomen. Ritsma et al. (2012) developed a window model for long term liver IVM (up to 1 month) to study liver metastasis. The window is composed of a titanium ring along with a 1mm groove. The window is secured on an abdominal wall by a purse-string suture in the groove and a coverslip is placed on the top for imaging window (Ritsma et al., 2012).

Liver IVM has been used to investigate liver transplantation, liver regeneration, and therapeutics of liver disease or injury (Theruvath et al., 2008; Rehman et al., 2011; Czerny et al., 2012; Liu et al., 2015, 2017; Krishnasamy et al., 2019; Wimborne et al., 2020). Moreover, the liver IVM model has also been used to study hepatic transport (Dunn and Ryan, 2017; Ryan et al., 2018; Tavakoli et al., 2019), flow modulation in liver microvasculature (Clendenon et al., 2019a, b), bile dynamic and (Meyer et al., 2017) and enzyme regulation. Furthermore, this model is also utilized to investigate liver during IR injury, infections, sepsis, and endotoxemia (Vollmar et al., 1997; McAvoy et al., 2011; Lu et al., 2014; Park et al., 2018).

#### Dorsal Skinfold Chamber Model

**FIGURE 3:**
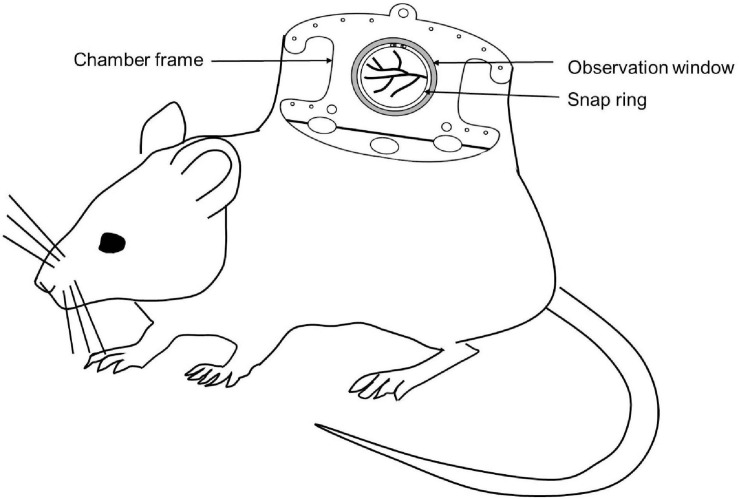
Schematic diagram of a mouse with a dorsal skinfold chamber for IVM.

The Dorsal skinfold chamber model is a widely used model for *in vivo* imaging. The chamber typically consists of two symmetrical metal frames. The frames contain a circular observation window. A double layer of depilated skin layer is sandwiched between these two frames. One of the layers of the skin along with subcutaneous tissue is removed completely in a circular area according to the diameter of the observation window. Then the circular coverslip is placed and fixed with the help of a snap ring (Figure 3). Titanium is the most commonly used metal to build the skinfold chamber but other varions from stainless steel or aluminum and non-metal materials are also used. The dorsal skinfold chamber model gives access to the striated muscle of the dorsal skin for IVM. After implanation, repetitive imaging can be performed up to 2–3 weeks (Schreiter et al., 2017; Dondossola et al., 2018; Hessenauer et al., 2018). It is suitable for both upright as well as inverted microscopes (Prunier et al., 2017).

The dorsal skinfold chamber has been extensively used in cancer biology to investigate tumor pathophysiology, tumor cell – microvasculature interaction, metastasis as well as therapy (Jain and Ward-Hartley, 1987; Jain et al., 2002; Alexander et al., 2013; Dondossola et al., 2018). It is also used to investigate the effect of chemical compounds on vascularization. Apart from that, the dorsal skinfold chamber model is also useful for studying interaction of biomaterials with surrounding host tissue, bacteria-endothelial cell interaction, organ transplantation, wound healing, fibrinolysis and thrombolysis, IR injury, inflammation, and sepsis (Laschke et al., 2011; Hillgruber et al., 2014; Miranda et al., 2015; Schreiter et al., 2017). The dorsal skinfold chamber is a widely accepted IVM model to investigate tissue angiogenesis and biocompatibility of biomaterials in tissue engineering.

#### Skull Cranial Window

The brain is the controlling unit of the entire body. Therefore, it is very important to understand brain physiology. *In vivo* imaging has become an important experimental tool to understand brain physiology and pathology. The brain is covered with a membrane known as the dura followed by two layers of compact cortical bone sandwiching a cancellous spongy bone layer (Yang et al., 2010; Zhao et al., 2018).

**FIGURE 4:**
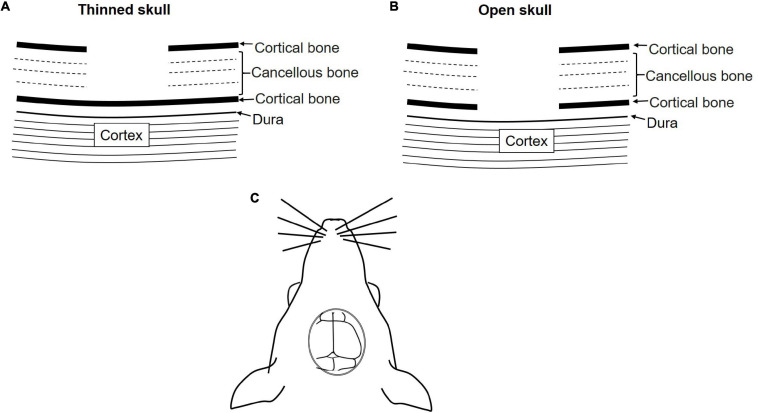
Schematic diagram of the skull cranial window for IVM. **(A)** Thinned-skull cranial window, **(B)** open-skull cranial window. **(C)** Dorsal view of the cranial window.

The brain can be accessed using two methods: open-skull or thinned-skull cranial window. As suggested by the name the thinned-skull cranial window is prepared by thinning of the skull bone layers with a drill. Controlled thinning is performed until the transparency for imaging is achieved without exposing the brain. On the other hand, in the open-skull window procedure, drilling is continued until all three bone layers are removed. A coverslip is positioned on the dura and sealed with adhesive (Figure 4) (Dorand et al., 2014; Isshiki and Okabe, 2014).

Both methods have their merits and demerits. Thinned-skull window approach causes minimal perturbation and allows immediate imaging after surgery whereas in open-skull model a resting period of approximately 2 weeks is required before imaging. Moreover, the open-skull method is prone to cause higher inflammation, astrogliosis, and higher dendritic spine turnover due to a higher degree of perturbation. Thinned-skull window approaches require re-thinning for repeated imaging, which is not necessary for open-skull approaches. Furthermore, image quality in the thin-skull window is compromised at points deeper than 50 μm. Therefore, the open-skull window is preferred for deep high resolution imaging. Depending on the requirement both models can be used for chronic as well as acute studies (Yang et al., 2010; Dorand et al., 2014). Both models require highly skilled surgeons and the selection of the model can be done based on the aim and the duration of the study.

Transcranial imaging can be used to study Alzheimer’s disease and potential treatments, brain injury, leukocyte-pathogen interaction and tumor dynamics in brain vasculature (Yang et al., 2010; Dorand et al., 2014; Isshiki and Okabe, 2014; Secklehner et al., 2017; Alieva et al., 2019).

## Discussion

Over the past decades, tissue engineering has made considerable progress in the field of tissue regeneration. Researchers are constantly applying novel approaches to understand tissue physiology in the normal and diseased state as well as regeneration or repair. 3D models closely resemble the native tissue environment. However, they cannot exactly mimic *in vivo* conditions where factors from the immediate and distant environment play an important role in maintaining tissue homeostasis (Kapałczyńska et al., 2018; Chen et al., 2019). Therefore, it is inevitable to perform *in vivo* studies. On the other hand, tissue repair or regeneration is a dynamic cellular process. Conventional *in vivo* studies are incapable to explain it thoroughly. Hence, performing IVM studies is the most appropriate approach for in depth understanding of tissue repair, regeneration, and cell interactions.

**FIGURE 5:**
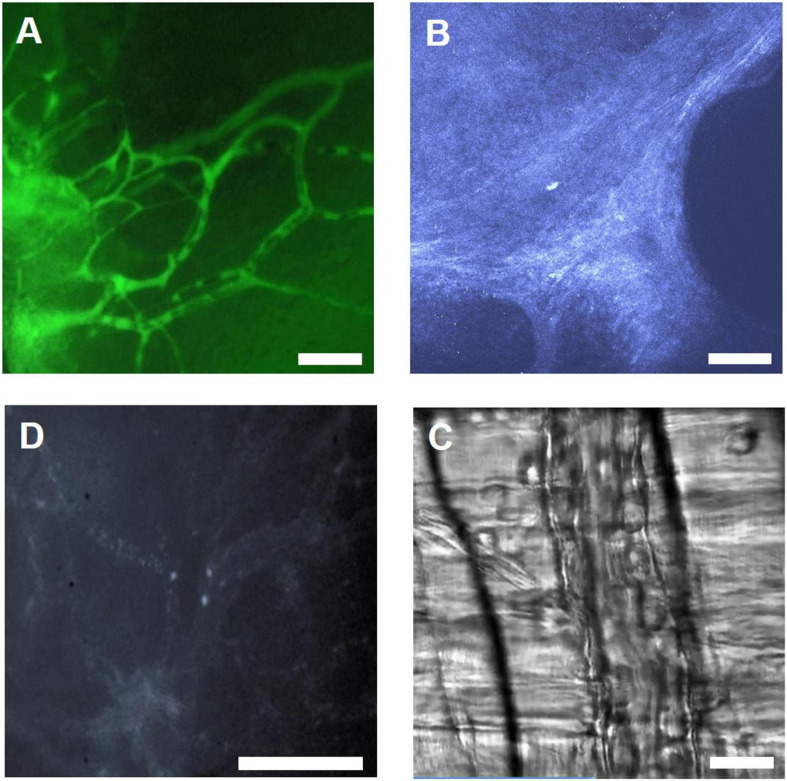
Illustration of IVM images using different microscopy methods. **(A)** IVM image of FITC-Dextran labeled microvasculature in the skinfold chamber, scale bar: 100 μm. **(B)** 2 PM THG image of collagen in the skinfold chamber, scale bar: 100 μm. **(C)** IVM image showing leukocytes labeled with Rhodamine 6G in the skinfold chamber, scale bar: 100 μm. **(D)** RLOT image of the cremaster muscle, scale bar: 50 μm.

Researchers have developed different IVM models, which require surgical procedures to expose the area of interest and install a window or chamber. Cremaster muscle and skinfold chamber models are the most preferable models in terms of simplicity and reproducibility to visualize capillary perfusion and leukocyte–endothelial interaction under native condition and under treatment as well as during IR injury (Figure 5). Cremaster muscle IVM model imaging is limited from few hrs up to 3 days (Siemionow and Nanhekhan, 1999). Therefore, the dorsal skinfold chamber model is widely preferred for the long-term *in vivo* imaging.

The dorsal skinfold chamber is a key model to analyze different tissue engineering strategies for improving the vascularization of implanted biomaterials. Long term, repetitive evaluation of the same ROI, evaluation of complex immunological phenomena is easily achieved. Kampmann et al. (2013) observed enhancement of PLGA scaffold vascularization upon application of Bone marrow-derived mesenchymal stem cells (bmMSCs) and VEGF. Reichel et al. (2015) investigated the effect of components of the Plasminogen Activation System on vascularization of porous polyethylene (PPE) implants. They observed accelerated vascularization in implants coated with urokinase-type plasminogen activator (uPA) and tissue plasminogen activator (tPA), plasminogen activator inhibitor-1 (PAI-1) (Reichel et al., 2015). Recently, improved vascularization of PPE implant coated with Vitronectin in skinfold chamber model was reported (Hessenauer et al., 2018). Adipose tissue-derived microvascular fragments (ad-MVF) contain high vascularization capacity that can be easily harvested from fat tissue. Frueh et al. (2017) seeded GFP^+^ ad-MVF on collagen-glycosaminoglycan matrices and implanted them into full-thickness skin defects in the mice skinfold chamber. They observed significantly accelerated vascularization of the implants (Frueh et al., 2017). This strategy can be used for full-thickness skin defect treatment. In another experiment, Laschke et al. (2019) cultivated ad-MVF at 20°C and implanted it into dorsal skinfold chambers. They observed enhanced vascularization in sub normothermically cultivated ad-MVFs compared to normothermically cultivated ad-MVFs (Laschke et al., 2019).

The skinfold chamber can also be adapted to investigate the regeneration of transplanted tissue material. Lushaj et al. (2012) implanted neonatal atrial and ventricular tissues in the skinfolds chamber. This was the first successful attempt of ectopic engraftment of differentiated myocardium in the skinfold chamber (Lushaj et al., 2012). In another experiment, Walser et al. (2013) implanted *in vitro* co-cultivated primary human osteoblasts and human dermal microvascular endothelial cells spheroids (HOB-HDMEC) into the skinfold chamber. They observed noticeable interconnection to the host microvasculature via the inosculation process. This strategy can be a very useful treatment of large bone tissue defects (Walser et al., 2013).

The dorsal skinfold chamber model is the most preferable model for investigation of biocompatibility and host reaction to different biomaterials. Jehn et al. (2020) compared the effects of mesenchymal stem cells (MSC) in combination with Poly-L-lactide-co-glycolide (PLGA) and beta-tricalcium phosphate (β-TCP) scaffold. They reported significant improvement in angiogenesis for β-TCP scaffolds compared with PLGA scaffolds (Jehn et al., 2020). Laschke et al. (2009) investigated *in vivo* biocompatibility and vascularization of porous polyurethane scaffolds. The scaffolds stimulated a weak angiogenic response after 14 days of implantation with low inflammatory reaction (Laschke et al., 2009). Gniesmer et al. (2020) studied chitosan-graft- polycaprolactone (CS-g-PCL) fiber mats for rotator cuff tear repair. Intravital investigation revealed significant increase in vascularisation in CS-g-PCL fiber mats compared to the porous polymer patch and uncoated PCL fiber mats on day 14 (Gniesmer et al., 2020). Dondossola et al. (2016) applied this model to examine foreign body response to 3D porous calcium phosphate-coated medical grade poly (ε-caprolactone) (mPCL-CaP) scaffolds. They observed a connection between giant cells and vascular endothelial growth factor (VEGF) induced neovessels as key factor stimulating the foreign body response and late-stage fibrosis (Dondossola et al., 2016). The same group also used a skinfold chamber model to study tumor-bone interactions and therapeutic response. They implanted tissue-engineered bone constructs in prostate cancer lesions. They observed tumor growth inside the bone cavity and along the cortical bone interface. They also reported reduction in osteoclast kinetics and osteolysis on application of bisphosphonate therapy without perturbing tumor growth (Dondossola et al., 2018).

Moreover, Polstein et al. (2017) used optogenetics for controlling cell differentiation and tissue formation in the skinfold chamber. They used a light-inducible switch to control the expression of angiopoietin-1 and VEGF for stimulation of vascular sprouting in a mouse dorsal skinfold chamber (Polstein et al., 2017).

In the aforementioned studies, repetitive evolution of the same ROI (region of interest) was performed to examine a comprehensive picture of the dynamic process starting from recruitment of cells to formation of a vasculature network or inosculation of host vasculature in scaffolds within the very same alive animal. This is only possible in IVM. Moreover, IVM enables observation of the immediate and long-term response of the native *in vivo* environment on the implantation of biomaterial in a live animal. The conventional approach for making similar observation requires termination of the experiment, extraction of the implant, and complex evaluation processes such as micro sectioning and staining. The harvesting and handling in this process itself can temper with final results. Imaging of dynamic cellular processes at multiple time points also reduces the requirement of total animals numbers for a particular study.

Skinfold chambers have also played an important role in cancer research and identification of key anti-tumor therapies. Yuan et al. (1995) used the skin fold chamber model to determine microvascular permeability in human tumor xenografts. Molecular size is one of the important determining factors for transvascular transportation of therapeutic agents in tumors. They concluded that liposomes of up to the diameter of 400 nm were permeable in human colon adenocarcinoma LS174T tumor vessels (Yuan et al., 1995). Vascular targeted therapies are showing promising results for cancer treatment. Several preclinical and clinical studies are reported which focus on blocking vasculature growth of the tumor. Savarin et al. (2018) monitored antiangiogenic or vascular disruptive effects of targeted gene and irradiation therapy from dorsal skin window. They observed a significant reduction in the tumor vessel area in animals receiving targeted gene treatment (Savarin et al., 2018). Haeger et al. (2019) determined that invading tumor cells survive DNA damage and radiotherapy via β1/αVβ3/β5 integrin crosstalk. Noticeably, effective radiosensitization can be accomplished by targeting multiple integrins (Haeger et al., 2019). Although, skin fold chamber is an excellent model for the cancer research, skin is not orthotopic location for all tumor types (Prunier et al., 2017).

The ability of reparative imaging provides an excellent opportunity to observe tumor cell metastasis and the effect of therapeutic treatment at several time points in real-time and in the same animal which is not possible in conventional cancer study designs (Condeelis and Segall, 2003).

Apart from the skinfold chamber, the skull cranial window is another important model for *in vivo* brain imaging. The skull cranial window model is more complex than the skinfold chamber. This model is very use full to study brain disease, injury and possible treatments (Yang et al., 2010; Dorand et al., 2014; Isshiki and Okabe, 2014; Secklehner et al., 2017; Alieva et al., 2019). Khosravi et al. (2018) used a cranial window chamber model to study angiogenesis and cellular events around surgical bone implants. Both cranial window and skinfold chamber models are widely used to study tumor development, cell–cell interaction, specific disease or injury and therapeutics (Upreti et al., 2011; Pai et al., 2014; Miranda et al., 2015; Reeves et al., 2015; Zhang et al., 2016; Ampofo et al., 2017; Tarantini et al., 2017; Alieva et al., 2019).

Intravital microscopy can be applied to study dynamic activities such as membrane trafficking. Endocytosis is a vital cellular process that plays an important role in the regulation of cell signaling, metabolism and motility. Moreover, the deregulation of the endocytic pathway is connected to infection, immunodeficiencies, neurodegeneration, and cancer (Ebrahim and Weigert, 2019). Molitoris group initiated an investigation of endosomal system dynamics in the kidney model. They applied multiphoton microscopy for *in vivo* imaging of uptake of systemically injected molecules such as fluorescent dextrans, folate receptors and albumin in the kidneys’ proximal tubuli (Dunn et al., 2002; Sandoval et al., 2004). Recently, Kuwahara et al. (2016) revealed Megalin as a potential therapeutic target for metabolic syndrome-related chronic kidney disease. The kidney IVM model is used to understand the function of different receptors present in the renal system. Schießl et al. (2016a) used intravital microscopy to investigate the effects of the angiotensin II (Ang II) receptor on podocyte function. They demonstrated that Ang II enhances the endocytosis of albumin by podocytes that can result in impaired podocyte function (Schießl et al., 2016a). The higher amount of albumin in urine or Albuminuria is an indication of the kidney disease. The salivary glands IVM model evolved as a relatively simple model for membrane trafficking studies (Ebrahim and Weigert, 2019). Secretory cells contain secretory granules, which transport a variety of proteins. Shitara et al. (2020) demonstrated the role of Cdc42 GTPase in the biogenesis and/or maturation of these secretory granules. Membrane remodeling is important for the regulation of different processes such as cell division, migration, and membrane trafficking that requires continuous modifications of the composition as well as the property of the lipid bilayer. Milberg et al. (2017) applied the salivary gland model to investigate the role of the actomyosin cytoskeleton in membrane remodeling. They reported that the actomyosin cytoskeleton serves as a scaffold for the recruitment of regulatory molecules and also provides necessary mechanical forces for remodeling the lipid bilayer (Milberg et al., 2017).

Hackl et al. (2013) used repeated MPM of the same glomeruli for imagining the motility of podocytes in the multi-color Pod-Confetti mouse model. They observed the appearance of a new podocyte within 24 h of the previous imaging session (Hackl et al., 2013). In a recent study, Schiessl et al. (2018) used MPM for evaluation of the cellular and molecular activities involved in renal proximal tubular regeneration. They observed proliferating tubular cells at the site of injury (Schiessl et al., 2018).

These above-mentioned dynamic portrayals of processes such as membrane trafficking and cellular motility are otherwise not possible in conventional study design without terminating the study at several timepoints.

The lung and heart are the most difficult organs for *in vivo* imaging because of continuous movement. Different methods for stabilization and software-based video editing methods are established (Presson et al., 2011; Matsuura et al., 2018). Lung IVM models are extensively used for imaging of immune cell trafficking, alveolar perfusion, and gaseous exchange. Oxygen uptake and carbon dioxide disposal is the primary function of the lung. Tabuchi et al. (2013) combined mice lung IVM along with two-dimensional oxygen saturation mapping to study pulmonary oxygen uptake. They demonstrated that 50% of total oxygen uptake takes place in precapillary arterioles of less than 30 μm in diameter before the blood enters the alveolar-capillary network (Tabuchi et al., 2013). In another similar study, they used the same methodology to study alveolar dynamics and local gas exchange in the healthy and diseased lung (Tabuchi et al., 2016). In a recent study, the role of neutrophils in a sepsis-induced lung injury model was investigated using combinations of fluorescent dyes and antibodies to differentiate leukocyte subsets. The acute lung injury decreased the functional capillary ratio due to the generation of dead space by prolonged neutrophil entrapment within lung capillaries (Park et al., 2019). Initial *in vivo* heart studies for leukocyte trafficking used heterotopic heart tissue transplantation due to inherent technical difficulties in imaging moving tissue (Li et al., 2012). Later Lee et al. (2012), introduced a two-photon method for intravital visualization of murine heart at subcellular resolution. Recently, novel cardiac stabilizers were established for imaging the beating native heart within the intrathoracic position in rats (Matsuura et al., 2018). They successfully managed real-time *in vivo* imaging of cardiac tissue dynamics under normal and IR conditions at subcellular resolution. They observed the subcellular dynamics of the myocardium and mitochondrial distribution in cardiac myocytes. They also observed IR injury induced suppression of the contraction/relaxation cycle and the resulting increase in cell permeability and leukocyte accumulation in cardiac tissue. Dynamics of immune cell trafficking immediately after events such as myocardial infarction is only possible in an IVM study design.

Liver IVM models have been used for the investigation of liver injury such as IR, and bacterial (*Mycobacterium bovis*, *Borrelia burgdorferi*, *acillus cereus*, and methicillin-resistant *Staphylococcus aureus*) and parasitic (*Plasmodium berghei*, *Leishmania donovani*, and *Schistosome granulomas*) infections and their treatment (Marques et al., 2015b). Acetaminophen is an antipyretic and analgesic drug. Recently, Hu et al. (2016) revealed that lower dosage of Acetaminophen induces reversible mitochondrial permeability resulting in mitochondrial dysfunction and steatosis in hepatocytes in the murine liver (Hu et al., 2016). Inhibition of the bile salt export pump (BSEP) is strongly connected to drug-mediated liver injury that commonly goes undetected during clinical testing. Ryan et al. (2018) used quantitative intravital microscopy to identify the dose-dependent effects of BSEP inhibitors. They used fluorescent bile salts as a biomarker for hepatobiliary transport inhibition. This model can provide valuable information on the toxic effects of the drug on human liver (Ryan et al., 2018). Dynamic processes such as cell–cell, cell–pathogen interaction, fluctuation in mitochondrial function, and bile transport can only be visualized in realtime using the IVM approach.

Imaging duration was one of the major limitations of IVM studies. Earlier models for abdominal organs were mainly acute. Ritsma et al. (2012) made a breakthrough in the area of the abdominal imaging window (AIW). They developed a window model for long-term liver IVM (up to 1 month) (Ritsma et al., 2012). Most of the current AIWs are designed and installed as described by Ritsma et al. (2012). Moreover, this window design can also be used to visualize internal organs such as the spleen, kidney, small intestine, pancreas, and liver. Perry et al. (2019) observed enhanced neovascularization and integration of pre-vascularized tissue-engineered muscle graft into abdominal wall defects compared to non-prevascularized grafts. Recently, Entenberg et al. (2018) developed a permanently implantable lung window that can be imagined for up to 2 weeks. Also, tissue regeneration is a dynamic and lengthy process that starts with the recruitment of immune cells following injury. Another imaging strategy for extension of the imaging period is the repeated externalization of organs or tissue (Hackl et al., 2013). However, surgical processes involved in repeated externalization of an organ can damage the organ of interest and delay the regeneration process or can lead to false results. Moreover, it is also prerequisite to keep organ or tissue wet and maintain the normal temperature during the surgical process. A precise experimental design is required for imaging of the entire regeneration process.

In most chamber or window models a glass coverslip is placed on the top of the tissue which can induce an inflammatory or immune response. Biocompatibility of the glass coverslip can be improved with PLL-g-PEG(poly-L-lysine-graft-poly(ethylene glycol)) coating (Ritsma et al., 2013). It is questionable if data collected from a small field can be extrapolated to the entire organ. Intravital microscopy provides high-resolution imaging of one small region that provides dynamic information of that spot only. For repeated analysis, it is important to identify the very same spot for the next microscopy. Hackl et al. (2013) used serial MPM imaging of the same glomerulus over time in the intact Pod-GFP mouse kidney. They identified Glomeruli based on a laser-induced mark placed close to the glomerulus (Hackl et al., 2013). For a better understanding of the dynamic process, imaging of more than 2 or 3 fields from a single animal is important. However, it can be difficult to fully correlate information because each field image contains small temporal heterogeneity. Here, it would be interesting to include a system that can collect data from multiple fields at the same time. However, imaging at lower magnification objectives such as 2.5× could be helpful because smaller magnification objectives provide a larger field of view than higher magnification objectives such as 25×. Though, lower magnification objectives can compromise the resolution (Dunn and Ryan, 2017).

The IVM studies are designed for imaging *in vivo* cellular dynamics. However, the surgical procedure involved during the experiment itself can interfere with the dynamics of cellular activity. Continuous exposure to light in different microscopic modalities is reported as phototoxic. Organs and tissues are a multicellular structural system. It is difficult to discriminate each cell type in one region. Here, *in vivo* imaging can be strengthened using different labeling strategies. Various approaches such as fluorescence dyes, fluorescence proteins and QDs are available. The specificity of these fluorescent probes can be increased using specific antibodies or antibody fragments. Discrimination of different cell types in one particular region can be achieved by combining one or more of the aforementioned strategies. For instance, GFP-positive animals can be injected with different cell marking dyes and marker antibodies at the same time (Dunn et al., 2002; Sandoval et al., 2004). Residual cell debris containing fluorescent proteins can result in unwanted background. Administration of all these substances can cause a toxic effect on the animal. Therefore, it is important to determine the optimal amount which exhibits minimum toxicity without interfering with the image quality. Moreover, some studies are designed for repeated *in vivo* imaging. Here, it is important to perform a preliminary study to determine the effect of long-term and repeated administration of these fluorescent probes.

From our own experience of animal studies and for both ethical and scientific reasons, it is very important to pay special attention to animal health. Animals need to be checked regularly for the overall health and healing of surgical areas. Many long-term windows or chamber installation requires placement of glass coverslips which are prone to break occasionally. Animals often tend to remove sutures placed to fix the chamber or window. Therefore, a regular check-up is necessary to prevent incidents that might affect the experiment outcome.

Penetration of depth is one of the major concerns for IVM studies. Conventional single-photon optical modalities such as epifluorescence and confocal microscopy can reach up to around 100 μm of depth only. Compared to conventional to one-photon confocal microscopy, 2 PM can improve the depth of penetration by a factor of 2 to 3 (Kobat et al., 2011). Theer et al. (2003) used a 800 nm excitation source by using a Ti: Sapphire regenerative amplifier. They could achieve 1 mm imaging depth in the mouse brain. Later, Kobat et al. (2011) used 1,280-nm excitation to achieve a remarkable penetration depth of approximately 1.6 mm in the cortex of a mouse brain. However, in 2 PM light scattering and absorption of tissue limit the penetration depth and both of these are dependent on excitation wavelength (Miller et al., 2017). In 2 PM microscopy, the highest imaging depth is determined by the ability of excitation light to hit the focus point unscattered as well as the released fluorescence to reach the detector (Kobat et al., 2011). Horton et al. (2013) developed a revolutionary system in the field of 3 PM. They used 3 PM to imaging of subcortical structures within an intact mouse brain. In 3 PM, 1,700 nm excitation source was used. Application of longer excitation wavelength reduces the attenuation of excitation light by the tissue. Moreover, 3PE significantly reduces the out-of-focus background and improves the signal to background ratio. In the preliminary 3PM experiments, vascular and neuronal structures in the mouse brain at ∼1.3 mm depth were imagined (Horton et al., 2013). Certainly, 3 PM has the potential to play a game-changing role in the field of IVM. Current 3 PM applications are largely limited to brain IVM (Horton et al., 2013; Wang T. et al., 2018). Application of innovative microscopic methods on other organs such as heart, lung, and kidney can achieve previously unmet penetration depth. Though, the establishment of an advanced imagining system requires more money and optimization initially, once established it can uncover dynamic activities deep inside the tissue that was hidden so far.

Overall, suitable selection and application of advanced optical modalities with fluorescence tagging methodologies in IVM can enable in-depth analysis of the tissue in healthy and diseased state, tissue development, repair and biomaterial compatibility as well as host reactions on implantation. It can also provide essential information at the level of cell–cell interactions and facilitate the development of potential treatments for complex diseases such as cancer and Alzheimer.

## Summary/Conclusion

Intravital microscopy provides information at the cellular and molecular level in different dynamic complex processes. It can be performed in both acute as well as chronic settings using windows or chambers. Advancement in microscopy and fluorescent markers have changed the direction of IVM. IVM provides useful information to understand physiology and cellular interaction. It can be applied to disease models for exploring new therapeutic approaches. Selection of the right model and suitable microscopic methods are very important points to be considered. Respiration and heart beating pose problems in imaging of upper extremity organs such as heart and lung. Deep tissue imaging is possible via multiphoton microscopy. However, there is still scope for development in further deep tissue imaging and application of advanced microscopic tool such as 3 PM for deep *in vivo* imaging of organs such as lung, liver, heart, kidney, and spleen.

Intravital microscopy is a promising approach to investigate host reactions on implanted biomaterials (Dondossola et al., 2016; Gniesmer et al., 2020; Jehn et al., 2020). IVM models for different organs have already been developed but most models are currently used to analyze organ specific dynamic processes during the healthy or diseased state. The majority of current IVM experiments can be adapted to improve tissue engineering strategies. IVM has great potential to improve and expand the boundaries of regenerative medicine. Considering all the advantages of IVM, it would be beneficial to keep developing and applying IVM models compatible with tissue engineering experiments in order to gain deeper insight in angiogenesis, inflammation and immunologic processes in tissue engineering.

## Author Contributions

RV, AA, RH, and MH wrote the manuscript. All authors contributed to the article and approved the submitted version.

## Conflict of Interest

The authors declare that the research was conducted in the absence of any commercial or financial relationships that could be construed as a potential conflict of interest.
